# Supplementary material for: The diagnostic accuracy of digital PCR, ARMS and NGS for detecting KRAS mutation in cell-free DNA of patients with colorectal cancer: A systematic review and meta-analysis
Source: PLoS One. 2021 Mar 26;16(3):e0248775. doi: 10.1371/journal.pone.0248775 (PMC7997033; doi:10.1371/journal.pone.0248775)
Supplement: S1 Table — (DOCX) [file pone.0248775.s001.docx]

| Database | Search strategy |
| --- | --- |
| Pubmed | ("KRAS") AND ("NGS" OR "next generation sequencing" OR "digital PCR" OR "ARMS" OR "amplification refractory mutation system") AND ("colorectal cancer" OR "colon cancer" OR "rectal cancer") AND ("ctDNA" OR "circulating tumor DNA" OR "cfDNA" OR "cell-free DNA" OR "liquid biopsy") |
| Embase | (KRAS.mp.) and (NGS.mp. or next generation sequencing.mp. or high throughput sequencing/ or digital PCR.mp. or digital polymerase chain reaction/ or ARMS.mp. or amplification refractory mutation system.mp.) and (colorectal cancer.mp. or colon carcinoma/ or rectum tumor/ or rectum carcinoma/ or colon tumor/ or colorectal cancer/ or rectum cancer/ or colon cancer/ or rectal cancer.mp. or rectum cancer/) and (ctDNA.mp. or circulating tumor DNA/ or cfDNA.mp. or cell-free DNA.mp. or liquid biopsy.mp. or liquid biopsy/) |
| Cochrane Library | ((cell-free DNA):ti,ab,kw OR (cfDNA):ti,ab,kw OR (circulating tumor DNA):ti,ab,kw OR (ctDNA):ti,ab,kw OR (liquid biopsy):ti,ab,kw) AND KRAS AND ((NGS):ti,ab,kw OR (next generation sequencing):ti,ab,kw OR (digital PCR):ti,ab,kw OR (ARMS):ti,ab,kw OR (amplification refractory mutation system):ti,ab,kw) AND ((colon cancer):ti,ab,kw OR (colorectal cancer):ti,ab,kw OR (rectal cancer):ti,ab,kw) |
